# Discovery of *mcr-1*-Mediated Colistin Resistance in a Highly Virulent Escherichia coli Lineage

**DOI:** 10.1128/mSphere.00486-18

**Published:** 2018-10-10

**Authors:** Brian M. Forde, Hosam M. Zowawi, Patrick N. A. Harris, Leah Roberts, Emad Ibrahim, Nissar Shaikh, Anand Deshmukh, Mazen A. Sid Ahmed, Muna Al Maslamani, Kyra Cottrell, Ella Trembizki, Lana Sundac, Heidi H. Yu, Jian Li, Mark A. Schembri, David M. Whiley, David L. Paterson, Scott A. Beatson

**Affiliations:** a School of Chemistry and Molecular Biosciences, The University of Queensland, Brisbane, Queensland, Australia; b Australian Infectious Diseases Research Centre, The University of Queensland, Brisbane, Queensland, Australia; c Australian Centre for Ecogenomics, The University of Queensland, Brisbane, Queensland, Australia; d The University of Queensland, UQ Centre for Clinical Research (UQCCR), Herston, Queensland, Australia; e College of Medicine, King Saud bin Abdulaziz University for Health Sciences, Riyadh, Saudi Arabia; f World Health Organization Collaborating Centre for Infection Prevention and Control, Riyadh, Saudi Arabia; g Gulf Cooperation Council Center for Infection Control, Riyadh, Saudi Arabia; h King Abdullah International Medical Research Centre, Riyadh, Saudi Arabia; i Pathology Queensland, Brisbane, Queensland, Australia; j Microbiology Division, Department of Laboratory Medicine and Pathology, Hamad Medical Corporation, Doha, Qatar; k Department of Neurosurgery, Hamad Medical Corporation, Weill Cornell Medical College in Qatar, Ar-Rayyan, Qatar; l Department of Infectious Diseases, Hamad General Hospital, Doha, Qatar; m Monash Biomedicine Discovery Institute, Department of Microbiology, Monash University, Victoria, Australia; n The Life Science Centre, Biology, School of Science and Technology, Örebro University, Örebro, Sweden; Antimicrobial Development Specialists, LLC

**Keywords:** *Escherichia coli*, antibiotic resistance, genome analysis

## Abstract

Resistance to last-line polymyxins mediated by the plasmid-borne mobile colistin resistance gene (*mcr-1*) represents a new threat to global human health. Here we present the complete genome sequence of an *mcr-1*-positive multidrug-resistant Escherichia coli strain (MS8345). We show that MS8345 belongs to serotype O2:K1:H4, has a large 241,164-bp IncHI2 plasmid that carries 15 other antibiotic resistance genes (including the extended-spectrum β-lactamase *bla*_CTX-M-1_) and 3 putative multidrug efflux systems, and contains 14 chromosomally encoded antibiotic resistance genes. MS8345 also carries a large ColV-like virulence plasmid that has been associated with E. coli bacteremia. Whole-genome phylogeny revealed that MS8345 clusters within a discrete clade in the sequence type 95 (ST95) lineage, and MS8345 is very closely related to the highly virulent O45:K1:H4 clone associated with neonatal meningitis. Overall, the acquisition of a plasmid carrying resistance to colistin and multiple other antibiotics in this virulent E. coli lineage is concerning and might herald an era where the empirical treatment of ST95 infections becomes increasingly more difficult.

**IMPORTANCE** Escherichia coli ST95 is a globally disseminated clone frequently associated with bloodstream infections and neonatal meningitis. However, the ST95 lineage is defined by low levels of drug resistance amongst clinical isolates, which normally provides for uncomplicated treatment options. Here, we provide the first detailed genomic analysis of an E. coli ST95 isolate that has both high virulence potential and resistance to multiple antibiotics. Using the genome, we predicted its virulence and antibiotic resistance mechanisms, which include resistance to last-line antibiotics mediated by the plasmid-borne *mcr-1* gene. Finding an ST95 isolate resistant to nearly all antibiotics that also has a high virulence potential is of major clinical importance and underscores the need to monitor new and emerging trends in antibiotic resistance development in this important global lineage.

## Abstract (precis)

Escherichia coli ST95 is a globally disseminated clone frequently associated with bloodstream infections and neonatal meningitis. However, the ST95 lineage is defined by low levels of drug resistance amongst clinical isolates, which normally provides for uncomplicated treatment options. Here, we provide the first detailed genomic analysis of an E. coli ST95 isolate that has both high virulence potential and resistance to multiple antibiotics. Using the genome, we predicted its virulence and antibiotic resistance mechanisms, which include resistance to last-line antibiotics mediated by the plasmid-borne *mcr-1* gene. Finding an ST95 isolate resistant to nearly all antibiotics that also has a high virulence potential is of major clinical importance and underscores the need to monitor new and emerging trends in antibiotic resistance development in this important global lineage.

## INTRODUCTION

Polymyxins B and E (colistin) have been used in veterinary and human medicine for over 50 years. They have broad-spectrum activities against Gram-negative bacteria and are effective against most Enterobacteriaceae. Unfortunately, colistin is associated with both nephrotoxicity and neurotoxicity (1), and due to these adverse effects, it has seen limited use in human medicine. However, colistin has now emerged as an effective therapeutic against carbapenem-resistant Enterobacteriaceae (CRE) (2), carbapenem-resistant Acinetobacter baumannii (CRAB) (3), and *Pseudomonas* species (4), for which treatment options are limited (5). This overreliance on colistin for treatment of these extensively resistant infections has seen the emergence of CRE, CRAB, and *Pseudomonas* isolates resistant to colistin (6–10). Colistin resistance is typically mediated by chromosomal mutations resulting in modifications to lipopolysaccharide (LPS), the target site of the polymyxins, and a reduction in polymyxin affinity. These chromosomal mutations are only vertically transmissible, and until recently, the polymyxins remained one of the last classes of antibiotic where resistance was not spread horizontally from cell to cell (9–12).

In 2015, Liu et al. described for the first time a plasmid-borne transmissible colistin resistance gene, *mcr-1* (13). The *mcr-1* gene belongs to the phosphoethanolamine transferase family of enzymes, which function by catalyzing the 4′-phosphoethanolamine (PEA) modification of lipid A, a component of LPS (13, 14). To date, *mcr-1* has been identified on both broad-host-range and narrow-host-range plasmids of different replicon types, including IncI2, IncX4, IncP, IncHI1, and IncHI2 (13, 15–17). Worryingly, carriage of *mcr-1* is often associated with cocarriage of other drug resistance genes, including those for carbapenemases (18–20) and extended-spectrum β-lactamases (18, 20, 21). This coassociation of *mcr-1* with other drug resistance genes is a significant step toward the emergence of pandrug resistance in the Enterobacteriaceae.

The *mcr-1* gene has been identified in a number of different bacterial species, but to date, carriage of *mcr-1* is most frequently associated with Escherichia coli (22). In a study of historical E. coli isolates in China, the emergence of the *mcr-1* gene was traced back to the 1980s, which coincides with the introduction of colistin as a growth enhancer in food production (23). The study, which screened 1,611 E. coli strains of chicken origin collected from farms in China between 1970 and 2014, found that the proportion of *mcr-1*-positive E. coli isolates increased exponentially from 5.2% in 2009 to 30% in 2014 (23). Similar proportions of *mcr-1*-positive E. coli strains have been observed in surveillance studies of food and production animals globally (13, 22, 24). The carriage rates of *mcr-1* are much lower in human E. coli isolates than in those from mammals and birds (13, 25). However, as extraintestinal pathogenic E. coli (ExPEC) strains that colonize humans and animals are highly similar (26–28), there is enormous zoonotic potential for mammals and birds to act as reservoirs of infection and transmit the *mcr-1* gene to humans (29, 30).

E. coli sequence type 95 (ST95) is a global pandemic clone of ExPEC. In contrast to other pandemic clones, such as E. coli ST131, ST95 isolates are characterized by a low incidence of multidrug resistance (MDR) (31–33). For example, ST95 clinical isolates had the lowest level of antibiotic resistance in comparisons among the 10 most prevalent uropathogenic E. coli (UPEC) sequence types (32, 33). Similar low levels of drug resistance have been identified in ST95 clinical isolates from the United States, Canada, and France (34–37). A highly virulent O45:K1:H7 subclone of ST95 accounts for one-third of all neonatal meningitis cases in France (38). A key feature of this subclone is a ColV-like virulence plasmid (39).

Here, we describe the first complete genome of an *mcr-1-*positive E. coli isolate (MS8345) of human origin. MS8345 was isolated from a patient in Qatar with a subarachnoid hemorrhage and was resistant to multiple antibiotics. We show that MS8345 contains two large plasmids associated with resistance and virulence, respectively, and is phylogenetically related to strains within a discrete clade in the ST95 lineage (E. coli phylogroup B2) that cause meningitis and severe avian infection. The acquisition of plasmid-borne colistin resistance in this highly virulent E. coli lineage is of major concern to global health.

## RESULTS

### Identification and characterization of an *mcr-1*-positive *E. coli* strain.

A single E. coli strain (0.37% of a total of 267 E. coli strains) from a diverse collection of Gram-negative bacterial pathogens in the Gulf Cooperation Council states of the Middle East was identified to possess the *mcr-1* gene. The strain, designated MS8345, was nonsusceptible to colistin, polymyxin B, multiple β-lactams (ampicillin, cefazolin, ceftriaxone, ceftazidime, cefepime), and most non-β-lactams (gentamicin, tobramycin, fluoroquinolones, trimethoprim, and trimethoprim-sulfamethoxazole). MS8345 was susceptible to meropenem, nitrofurantoin, the β-lactamase cefoxitin, and to the β-lactam/β-lactamase inhibitors amoxicillin-clavulanic acid and piperacillin-tazobactam (Table 1).

**TABLE 1:** MICs of antibiotics for MS8345^*a*^

Antimicrobial	MIC (mg/liter)	Interpretation	Gene
Ampicillin	≥32	R	*TEM-1B*
Amoxicillin-clavulanic acid	8	S	NA
Ticarcillin-clavulanic acid	32	R	
Piperacillin-tazobactam	≤4	S	NA
Cefazolin	≥64	R	*CTX-M-1*
Cefoxitin	≤4	S	NA
Ceftazidime	4	R	*CTX-M-1*
Ceftriaxone	≥64	R	*CTX-M-1*
Cefepime	2	R	*CTX-M-1*
Colistin	8^*b*^	R	*mcr-1*
Meropenem	≤0.25	S	NA
Amikacin	≤2	S	NA
Gentamicin	≥16	R	*aac(3)-IIa*
Tobramycin	4	R	*aac(3)-IIa*
Ciprofloxacin	≥4	R	*gyrA*
Norfloxacin	8	R	*gyrA*
Nitrofurantoin	≤16	S	NA
Trimethoprim	≥16	R	*drfA*
Trimethoprim-sulfamethoxazole	≥320	R	*drfA*
Polymyxin B	4^*b*^	R	*mcr-1*

a R, resistant; S, susceptible; NA, not applicable.

b Tested by broth microdilution; all other MICs were obtained with Vitek2.

### MS8345 is closely related to a highly virulent clonal lineage associated with neonatal meningitis.

**FIG 1:**
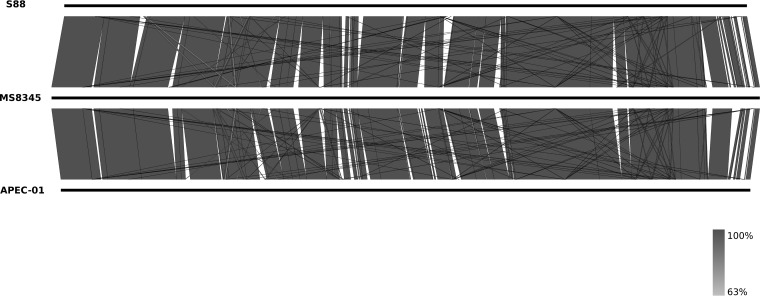
Pairwise whole-genome nucleotide comparison of E. coli MS8345, S88, and APEC-O1. Black bars represent the chromosome of each strain, and the gray shading represents regions of nucleotide sequence identity (63% to 100%) determined by BLASTn analysis. The figure was prepared using Easyfig (66).

**FIG 2:**
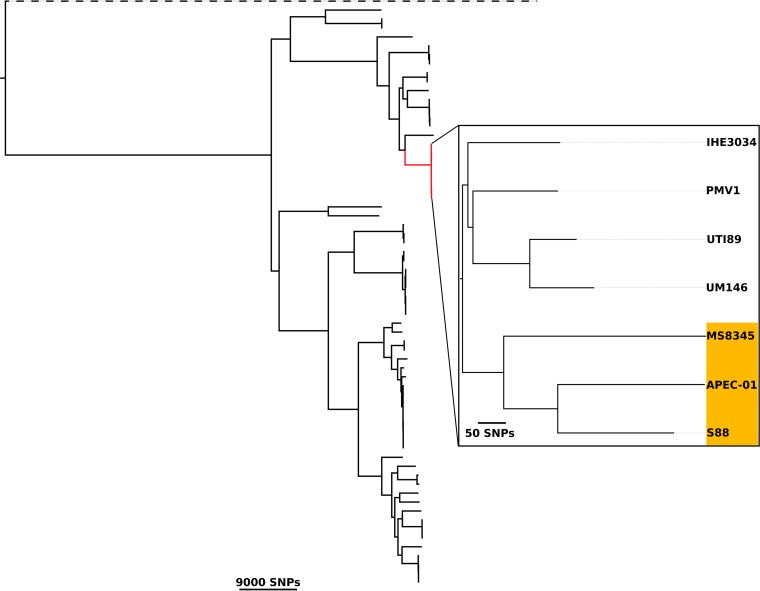
Phylogenetic tree of ST95 E. coli isolates. Maximum-likelihood phylogenetic tree of MS8345 and 65 additional complete E. coli chromosomes built using 111,631 core single-nucleotide polymorphisms (SNPs). The ST95 clonal complex is highlighted in red. The enlarged area displays a high-resolution maximum-likelihood phylogenetic tree of 7 ST95 isolates grouped into two discrete subclades in which MS8345 clusters with S88 and APEC-O1. Strains carrying a Col-like (ColV or ColBM) virulence plasmid are highlighted in orange. The tree was rooted using Escherichia fergusonii (dashed branch). Scale bars indicate branch lengths in numbers of SNPs. Phylogeny was visualized using FigTree (http://tree.bio.ed.ac.uk/software/figtree/).

**FIG 3:**
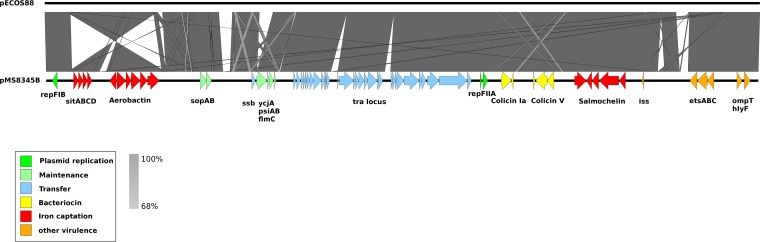
Pairwise nucleotide comparison of the ColV-like virulence plasmids pS88 and pMS8345B. Protein-coding genes involved in replication, plasmid transfer, maintenance, and virulence are represented by colored arrows (as indicated in the key). The results of a BLASTn comparison between the two plasmids are shown with gray or yellow for (reverse-complement) shading between 68% and 100% nucleotide identities. Yellow indicates a match. Several large insertion or deletion events are evident between both plasmids as is an inversion in MS8345 that reversed the order of the *sitABCD* and aerobactin genes.

The complete genome of E. coli MS8345 comprised a single circular chromosome 5,220,996 bp in length with an average G+C content of 50.5% and two circular plasmids: a 241,164-bp multidrug resistance (MDR) plasmid (pMS8345A) containing *mcr-1* and a 133,283-bp virulence plasmid (pMS8345B). *In silico* multilocus sequence typing (MLST) identified MS8345 as ST95. MS8345 was serotyped as O2:K1:H4 and possesses the *fimH*27 allele, placing it in the recently defined ST95 subgroup E (40). Pairwise genome comparisons revealed MS8345 to be highly similar to the human neonatal meningitis E. coli (NMEC) isolate S88 (GenBank accession number CU928161) and the avian pathogen APEC-O1 (GenBank accession number CP000468) (Fig. 1). Phylogenetic analysis demonstrated clustering of MS8345 with S88 and APEC-O1 in a clade discrete from the other completely sequenced ST95 strains (Fig. 2). The majority of ST95 complete genomes do not contain MDR plasmids, but several have been found to harbor a virulence plasmid (Fig. 2). Plasmid pMS8345B is highly similar to the ColV-like virulence plasmid pS88 (GenBank accession number CU928146) from E. coli S88 and carries an identical complement of virulence factors and iron uptake systems, specifically, *etsABC*, *ompT, hlyF*, the *sitABCD* operon, salmochelin (*iroBCDEN*), and aerobactin (*iucABCD* and *iutA*) (Fig. 3) (39).

### The *mcr-1* colistin resistance gene is borne on a large MDR IncHI2 plasmid.

**FIG 4:**
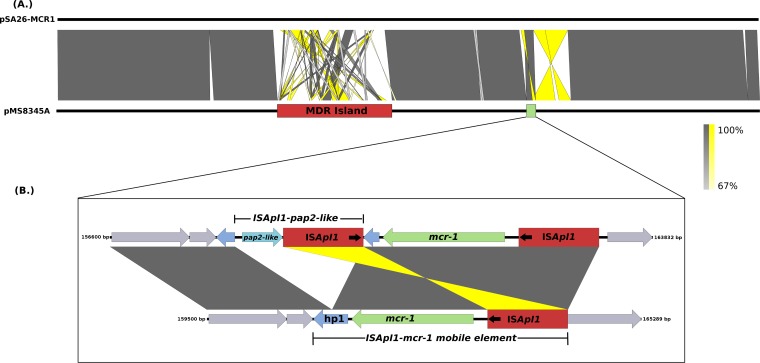
Pairwise nucleotide comparison of *mcr-1*-positive multidrug resistance plasmids pSA26-MCR1 and pMS8345. (A) A comparison of *mcr-1*-positive plasmids from Qatar and Saudi Arabia reveal them to have highly similar backbones. However, both plasmids differ considerably in their resistance gene complement, clearly visible as a large, highly variable MDR region characterized by numerous insertions, deletions, inversions, and rearrangements. The MDR island is represented by a red rectangle and the *mcr-1* region by a green rectangle. (B) Comparison of the IS*Apl1-mcr-1* mobile elements from pMS8345A and pSA26-MCR1. IS*Apl1* mobile elements are represent by red rectangles. Black arrows indicate the orientation of the insertion sequence (IS) elements, and protein-coding regions are represented by the colored arrows.

The *mcr-1* gene was located on pMS89345A, a 241-kb IncHI2 plasmid sharing high sequence identity (99% nucleotide identity, 89% sequence length) with the *mcr-1-*positive plasmid pSA26-MCR1 (GenBank accession number KU743384). pSA26-MCR1 was identified in the carbapenem-resistant (*bla*_NDM-1_-positive) E. coli ST68 strain SA26 isolated in Saudi Arabia and is highly similar (99% nucleotide identity, 84% sequence length) to the IncHI2 *mcr-1*-positive plasmid pHNSHP45-2 (GenBank accession number KU341381) from the Chinese pig isolate SHP45 (41) and to *mcr-1*-negative plasmids carried in human, poultry, and pig *Salmonella* isolates from China (99% nucleotide identity, 86% sequence length) (15, 41). The major difference between pMS8345A and pSA26-MCR1 is the structure and content of a large MDR region carried on both plasmids (coordinates 74,782 to 112,611 and 73,858 to 112,218, respectively). The MDR region of pMS8345A contains eight resistance genes that are not present in the MDR region of pSA26-MCR1 and includes a single copy of *bla*_CTX-M-1_ carried on an IS*Ecp1* mobile element. In pMS8345A and pSA26-MCR1, *mcr-1* is not a component of the MDR region, but instead, the *mcr-1* mobile element IS*Apal1-mcr-1* (42) is inserted 46,815 bp and 47,761 bp downstream (Fig. 4A). The IS*Apal1-mcr-1* elements of pMS8345A and pSA26-MCR1 are identical (100% nucleotide sequence identity); however, in pSA26-MCR1, the gene encoding the hypothetical protein hp1, downstream of *mcr-1*, has been disrupted by insertion of a second IS*Apl1* element carrying a putative pap2-like phosphatase (15) (Fig. 4B). No additional *mcr*-type genes were identified.

### Additional resistance genes.

The MDR island of pMS8345A carries 15 additional resistance genes and encodes three putative multidrug efflux pumps, which together provide resistance to multiple classes of antibiotics, including aminoglycosides, β-lactams, macrolides, sulfonamides, tetracycline, and trimethoprim. In addition, a further 14 resistance genes were identified on the MS8345 chromosome. Chromosomally borne resistance genes were distributed across four discrete locations and included two nearly identical copies of an ∼50-kb genomic island (GI) (53,078 bp and 52,977 bp, respectively) bearing *sul1*, *aadA1*, and *erm*(B). Finally, mutations in *gyrA* (resulting in S83L and D87N) and *parC* (S80I) that lead to fluoroquinolone resistance were also identified. *In silico* resistance profiling of MS8345 correlates with the observed phenotypic resistances reported via Vitek (Table 1). A complete list of resistance genes carried by MS8345 is reported in Table 2.

**TABLE 2:** Antibiotic resistance genes identified in *E. coli* MS8345

Gene	Locus tag	Requirement(s) for resistance phenotype	Location	Coordinates	Resistance to antibiotic(s)
*gyrA*	MS8345_02349	S83L D87N	Chromosome	2417780–2420407	Fluoroquinolones
*sul1*	MS8345_03203		Chromosome	3283535–3284374	Sulfonamide
*aadA1*	MS8345_03205		Chromosome	3284879–3285658	Spectinomycin, streptomycin
*erm*(B)	MS8345_03209		Chromosome	3288275–3289012	Erythromycin
*parC*	MS8345_03375	S80I	Chromosome	3452849–3455107	Fluoroquinolones
*bla*_TEM–1B_	MS8345_04039		Chromosome	4136995–4137855	Penicillin
*sul2*	MS8345_04043		Chromosome	4141072–4141887	Sulfonamide
*strA*	MS8345_04044		Chromosome	4141948–4142751	Aminoglycosides
*strB*	MS8345_04045		Chromosome	4142751–4143587	Aminoglycosides
*tetR*	MS8345_04057		Chromosome	4151677–4152354	Tetracycline
*tetA*	MS8345_04061		Chromosome	4152433–4153632	Tetracycline
*dfrA1*	MS8345_04149		Chromosome	4244602–4245075	Trimethoprim
*aadA1*	MS8345_04151		Chromosome	4245752–4246540	Spectinomycin, streptomycin
*erm*(B)	MS8345_04728		Chromosome	4844585–4845322	Erythromycin
*aadA1*	MS8345_04732		Chromosome	4847939–4848718	Spectinomycin, streptomycin
*sul1*	MS8345_04733		Chromosome	4849219–4849887	Sulfonamide
*ampC*	MS8345_04780		Chromosome	4888466–4889599	Cephalosporins
*arr-2*	MS8345_A00270		pMS8345A	76547–76999	Rifampin
*ere(A)*	MS8345_A00271		pMS8345A	77275–78501	Erythromycin
*aadA1*	MS8345_A00272		pMS8345A	78587–79378	Spectinomycin, streptomycin
*bla*_CTX-M-1_	MS8345_A00277		pMS8345A	83441–84274	β-Lactams
*aac(3)-Iia*	MS8345_A00004		pMS8345A	87010–87870	Aminoglycosides
*tmrB*	MS8345_A00005		pMS8345A	87883–88425	Tunicamycin
*strA*	MS8345_A00011		pMS8345A	91706–92509	Aminoglycosides
*strB*	MS8345_A00012		pMS8345A	92509–93345	Aminoglycosides
*bla*_TEM-1B_	MS8345_A00015		pMS8345A	94194–95054	Penicillin
*mph(A)*	MS8345_A00018		pMS8345A	96389–97294	Macrolides
*sul1*	MS8345_A00025		pMS8345A	102605–103444	Sulfonamide
*dfra7*	MS8345_A00027		pMS8345A	104015–104716	Trimethoprim
*tetX*	MS8345_A00031		pMS8345A	107415–108551	Tetracycline
*sul1*	MS8345_A00032		pMS8345A	108707–109546	Sulfonamide
*aadA1*	MS8345_A00034		pMS8345A	110050–110904	Spectinomycin, streptomycin
*mcr-1*	MS8345_A00099		pMS8345A	160818–162443	Colistin, polymyxin B

## DISCUSSION

The recent discovery of the transmissible, plasmid-borne colistin resistance gene *mcr-1* poses a significant threat to global human health. Since the *mcr-1* gene was first identified in China in 2015 (13), multidrug-resistant human and animal bacterial isolates carrying the *mcr-1* gene have been reported in over 25 countries throughout Asia, Europe, the Middle East, North Africa, and North America (22). Here, we describe the first report of *mcr-1*-mediated colistin resistance in an ExPEC strain of the pandemic ST95 complex from the Persian Gulf region.

Genomic analysis of MS8345 revealed that it is highly similar to the neonatal meningitis strain S88 and the avian pathogenic strain APEC-O1. Notably, these three strains are phylogenetically more related to one another than to other ST95 strains for which a complete genome is available. S88 is a representative of the highly virulent O45:K1:H7 clone, which accounts for one-third of all neonatal meningitis cases in France (38) and carries a ColV-like virulence plasmid (pS88) (39) almost identical to plasmid pMS8345B in MS8345. Although MS8345 was isolated from respiratory secretions and attempts to culture it from the patient’s bloodstream and cerebrospinal fluid were unsuccessful, the extraordinary similarity between these two strains, despite their different O and H types, suggests that they may be equally virulent.

A key feature of the ST95 lineage is the low frequency of MDR among clinical isolates. In England, in a survey of the nine most common uropathogenic E. coli ST clonal lineages, ST95 clinical isolates were identified as having the lowest levels of resistance (32, 33). Similar low levels of drug resistance have been identified in ST95 clinical isolates from the United States, Canada, and France (34–37). However, despite low levels of resistance, ST95 remains a significant cause of extraintestinal E. coli infections worldwide. Low levels of antibiotic resistance provide for uncomplicated treatment options, but increasing levels of resistance among the ST95 strains pose a significant risk for treatment failure. Consequently, the emergence of ST95 isolates resistant to multiple antibiotics, including β-lactams and carbapenems, is worrying (37). In this study, *in silico* antimicrobial resistance profiling of MS8345 revealed it to carry 31 resistance genes, an unusually high number for this clone compared to the number carried by other ST95 strains, which typically have fewer than five resistance genes and remain susceptible to most antibiotics (31–33). Multidrug resistance in MS8345 is attributed to a large complement of acquired resistance genes carried on the chromosome and on the MDR plasmid pMS8345A. High levels of resistance, combined with an extensive virulence profile, characterized MS8345 as a significant outbreak threat and a likely reservoir of plasmid-mediated *mcr-1* trafficking in clinical environments and in the community, emphasizing the need for continuing surveillance.

Carriage of *mcr-1* in human isolates has so far been rare, with less than 2% of clinical Enterobacteriaceae isolates in China and ≤0.2% of clinical E. coli isolates in Europe testing positive (13, 25). Low rates of *mcr-1* carriage in human isolates might reflect the traditionally low levels of colistin usage in hospitals. In contrast, colistin is widely used to control diarrheal diseases in poultry and pigs (43). In 2018, the use of colistin in veterinary products is estimated to increase by ∼500% from 1992 usage levels, with China being the largest user, consuming an estimated 12,000 tonnes in 2015 (13, 22). High rates of colistin use in animal production is almost certainly a strong driver of selective pressure for colistin resistance in animal isolates. Indeed, the rate of *mcr-1* carriage in animals and in animal meat products is significantly greater than carriage rates in human isolates (22). For example, in a survey of three chicken farms in Tunisia, up to 83% of birds were estimated to be *mcr-1* positive (24). Notably, poultry and retail chicken meat are recognized as reservoirs of ExPEC in humans (30, 36, 44). A study of serogroup O45 ST95 ExPEC from Spain found human and avian isolates to be highly homogeneous (30), and a study of serotype O1, O2, and O18 APEC strains in China showed that APEC O1:K1 and O2:K1 strains are a major cause of colibacillosis in domestic and wild birds (35, 39, 45, 46) and can cause septicemia and meningitis in mammalian infection models (47). Here, we have shown that one of the closest relatives of MS8345 is the E. coli avian pathogen APEC-O1, highlighting the zoonotic potential of these bacteria and the potential impact of continued antibiotic misuse in animal production.

Recent reports describing *mcr-1*-positive plasmids from Enterobacteriaceae have shown that the IS*Apal1-mcr-1* element contains a putative *pap2* gene located immediately downstream of *mcr-1* (hypothetical protein MS8345_A200 in pMS8345A) (48, 49). The PAP2-like family of phosphatases is capable of modifying lipid A by replacing the negatively charged phosphate groups with a positively charged amine group (50). Changing the charge on LPS is a recognized strategy employed by some bacteria to increase resistance to cationic antimicrobial peptides, such as the polymyxins (51). Although MS8345_A200 does display some homology to putative PAP2 family proteins from other species (BLASTp, 86% amino acid identity, 55% query coverage), it does not possess any functional domains associated with this superfamily and at best represents a nonfunctional fragment of *pap-2*. However, over the course of our study, we identified an intact *pap2-like* gene associated with an IS*Apal1* element (IS*Apal1–pap2-like*), carried on the *mcr-1*-positive plasmid pA26-MCR1, which has inserted into a hypothetical protein highly similar to MS8345_A200 (15). Whether this *pap2-like* gene (unannotated, coordinates 158363 to 158905) is functional is currently unknown; however, its potential impact on colistin sensitivity provides an intriguing avenue for further research.

In summary, we provide the first report of an *mcr*-1-positive isolate of the E. coli ST95 lineage. Using long-read sequencing data enabled us to resolve the complete genome sequence, including the precise context of *mcr-1* on an IncHI2 plasmid and the resistance gene profile across three MDR genomic regions. The emergence of colistin resistance in this highly virulent ExPEC lineage is of serious concern to global human health.

## MATERIALS AND METHODS

### MCR-1 real-time PCR screening.

A total of 694 isolates, comprising Acinetobacter baumannii (*n =* 130), Klebsiella pneumoniae (*n* = 162), E. coli (*n =* 267), Pseudomonas aeruginosa (*n =* 128), Citrobacter freundii (*n* = 3), and Enterobacter cloacae (*n =* 4) isolates mainly from the Gulf Cooperation Council states of the Middle East, were tested by PCR. Isolates were prepared using a simple heat denaturation step to release nucleic acids, as previously described (52). Heat-denatured suspensions were pooled (10 isolates per pool) for PCR testing. Isolate pools were then simultaneously tested by two different real-time PCR assays: one using the QuantiTect SYBR Green PCR kit (Qiagen, Australia) as the basis for the reaction mix with previously described primers (CLR5-F and CLR5-R) (13) and the other using the QuantiTect Probe PCR kit with primers (ACAATCTCGGCTTTGTGCTGA and CGATACGATGATAACAGCGTGGT) and a TaqMan probe (FAM-TGCTCTTTGGCGCGATGCTACT-DQ, where FAM is 6-carboxyfluorescein and DQ is dark quencher) designed as part of this study. PCR assays were run simultaneously and returned identical results. All isolates from any pool providing a positive result were then tested individually. From this screening exercise, we identified a single E. coli isolate (MS8345) that contained the *mcr-1* gene.

### Case record.

MS8345 (also designated HZ-QTR-HMC-19) is an extended-spectrum β-lactamase (ESBL)-producing E. coli strain isolated from respiratory secretions of a 58-year-old male of Nepali origin, admitted to an intensive-care unit in Qatar with subarachnoid hemorrhage. His admission was complicated by hydrocephalus, requiring insertion of an extraventricular drain, and severe sepsis with multiorgan dysfunction following gastrointestinal perforation. However, he had no clear signs of ventilator-associated pneumonia at the time that MS8345 was isolated. During his admission, he had exposure to vancomycin, piperacillin-tazobactam, meropenem, and caspofungin but did not receive any treatment with polymyxins. He survived and was repatriated after 2 months in the hospital.

### Antibiotic resistance phenotypic testing.

Susceptibility testing to 18 different antibiotics was performed using automated broth microdilution (Vitek 2 card AST-N246; bioMérieux), and the presence of ESBL production was confirmed by clavulanate synergy with ceftriaxone and ceftazidime according to EUCAST standards. MICs of colistin (Beta Pharma Co. Ltd., Shanghai, China) and polymyxin B (Beta Pharma Co. Ltd.) were determined using broth microdilution according to the CLSI guideline. Twofold dilutions of colistin and polymyxin B ranging from 0 to 128 mg/liter were made in cation-adjusted Mueller-Hinton broth (CAMHB) (Oxoid, Hampshire, UK). Bacterial suspensions were prepared by suspending colonies from nutrient agar (School of Biomedical Sciences Media Unit, Monash University, Australia) in normal saline to match a 0.5 McFarland standard (DensiCHEK; bioMérieux). The suspensions were further diluted in CAMHB to yield ∼10^6^ CFU/ml. Antibiotic solution (100 µl) was added to 100 µl of diluted inoculum in 96-well microtiter plates (Techno Plas, St. Marys, South Australia), and MICs were determined at 20 h after incubation at 37°C. Quality control using P. aeruginosa ATCC 27853 was included as recommended by the CLSI.

### Genome sequencing and assembly.

Genomic DNA (gDNA) from MS8345 was sequenced on a PacBio RSII instrument (The Doherty Institute for Infection & Immunity, The University of Melbourne) using a single SMRT cell, a 15-kb insert library, and the P6 polymerase and C4 sequencing chemistry. *De novo* assembly of the raw PacBio sequencing data were done using the hierarchical genome assembly process (HGAP version 2) and quiver (53) from the SMRT Analysis software suite (version 2.3.0 [http://www.pacb.com/devnet/]) with default parameters. Following *de novo* assembly, the completeness of the chromosome and plasmids was visually verified using Contiguity (https://github.com/mjsull/Contiguity) (54). The complete chromosome and plasmids were then subjected to a polishing phase, during which the raw PacBio sequencing reads were mapped back onto the assembled circular contigs (BLASR [55] and quiver) to validate the assembly and resolve any remaining errors. gDNA from MS8345 was also prepared as Nextera XT libraries and sequenced on an Illumina NextSeq sequencer at the Australian Centre for Ecogenomics. The raw Illumina sequencing reads were used to resolve 1,046 single-nucleotide insertion and deletion errors associated with homopolymer tracts. Illumina reads were aligned to the complete genome of MS8345 using bwa version 0.7.12 (56), and a corrected consensus was called using Pilon version 1.18 (57).

### Multilocus sequencing typing.

*In silico* sequencing typing was performed using MLST version 2.8 (https://github.com/tseemann/mlst) and the E. coli typing scheme available from PubMLST (https://pubmlst.org/) (58).

### *In silico* serotyping.

Determination of O and H antigens was performed using SerotypeFinder version 1.1 (59). K antigen was determined using Kaptive (60) and an in-house database of E. coli capsule genes.

### Genome annotation and comparative genomics.

*In silico* functional annotation of MS8345 was performed using prokka (Prokka, Prokaryotic Genome Annotation System, http://vicbioinformatics.com/). Identification of antimicrobial resistance genes was performed using ResFinder version 2.0 (61). Additional screening for antimicrobial resistance genes was performed by screening the raw Illumina reads against the ARG-ANNOT database (62) using srst2 (63), and chromosomal genes associated with antibiotic resistance were manually inspect for point mutations known to contribute to a resistance phenotype (e.g., *gyrA*). Chromosome and plasmid comparisons were performed using BLASTn (64), the Artemis comparison tool (65), and Easyfig (66).

### Phylogeny.

MS8345 belonged to ST95. To determine the phylogenetic relationship of MS8345 to other ST95 isolates, we carried out phylogenomic analysis. Briefly, the complete genomes of MS8345, UTI89 (GenBank accession number CP000243), S88 (GenBank accession number CU928161), APEC-O1 (GenBank accession number CP000468), IHE3034 (GenBank accession number CP001969), PMV1 (GenBank accession number HG428755), UM146 (GenBank accession number CP002167), and other E. coli isolates were aligned using Parsnp (67). Recombinant regions were filtered from the alignment using Gubbins v2.1.0 (68), and core single-nucleotide polymorphisms (SNPs) were determined. A maximum-likelihood tree was estimated using RAxML (69) under the GTRGAMMA nucleotide substitution rate model.

### Ethics approval.

The permission for publication was granted by the Medical Research Centre at HMC (reference number MRC/0765/2017).

### Accession number(s).

The complete genome of MS8345 (chromosome and plasmids) has been deposited in GenBank under the accession numbers CP025401, CP025402, and CP025403. PacBio and Illumina sequence read data have been deposited in the Sequence Read Archive (SRA) under the accession numbers SRR6364639 and SRR6364638, respectively.
